# Symptomatic internal carotid artery dissection and kinking in a patient with fibromuscular dysplasia

**DOI:** 10.1590/1677-5449.200243

**Published:** 2021-05-31

**Authors:** Victor Bilman, Luca Apruzzi, Domenico Baccellieri, Francesca Sanvito, Luca Bertoglio, Roberto Chiesa

**Affiliations:** 1 “Vita – Salute” University, Scientific Institute H. San Raffaele, Milan, Italy.

**Keywords:** carotid artery, kinking, coiling, arterial dissection, fibromuscular dysplasia, artéria carótida, acotovelamento, enrolamento, dissecção arterial, displasia fibromuscular

## Abstract

Isolated dissection of the internal carotid artery (ICA) is rare in young patients and is a cause for strong suspicion of fibromuscular dysplasia (FMD), especially when associated with artery elongation and tortuosity. The natural history of cerebrovascular FMD is unknown and management of symptomatic patients can be challenging. We report the case of a 44-year-old female patient with a history of transient ischemic attack in the absence of cardiovascular risk factors, associated with an isolated left ICA dissection and kinking. Carotid duplex ultrasound confirmed the diagnosis of dissection and demonstrated severe stenosis of the left ICA. The patient underwent surgical repair and histopathological evaluation confirmed the diagnosis of FMD with dissection. An autogenous great saphenous vein bypass was performed and the patient had an uneventful recovery. Cervical carotid artery dissection can be related to underlying arterial pathologies such as FMD, and the presence of ICA tortuosity highlights certain peculiarities for optimal management, which might be surgical.

## Resumo

A dissecção isolada da artéria carótida interna em pacientes jovens é rara, e a displasia fibromuscular deve ser altamente suspeitada principalmente quando estiver associada a alongamento e tortuosidade da artéria. A história natural da displasia fibromuscular cerebrovascular é desconhecida, e o manejo de pacientes sintomáticos pode ser desafiador. Apresentamos o caso de uma paciente de 44 anos com histórico de ataque isquêmico transitório sem fatores de risco cardiovasculares, associado a dissecção e acotovelamento isolados da artéria carótida interna esquerda. O ultrassom duplo das carótidas confirmou o diagnóstico de dissecção e demonstrou estenose grave na artéria carótida interna esquerda. A paciente foi submetida a reparo cirúrgico, e a avaliação histopatológica confirmou o diagnóstico de displasia fibromuscular com dissecção. Foi realizada cirurgia de ressecção do segmento e reconstrução com veia safena magna autógena, e a paciente se recuperou sem complicações. A dissecção da artéria carótida cervical pode estar relacionada a doenças arteriais subjacentes, como a displasia fibromuscular, e a presença da tortuosidade da artéria carótida interna destaca algumas particularidades no manejo ideal, o qual pode ser cirúrgico.

## INTRODUCTION

Isolated carotid artery elongation with coiling or kinking in the absence of atherosclerotic lesions is a rare condition and the correlation between carotid tortuosity and symptomatic cerebrovascular disease is still questionable.^1^^,^^2^ Fibromuscular dysplasia (FMD) might predispose to internal carotid artery tortuosity and, especially in case of extracranial carotid dissection, can be associated with cerebrovascular symptoms including headache, pulsatile tinnitus, transient ischemic attack (TIA), or stroke.^3^^-^6 According to the United States (US) FMD registry, 18.3% of strokes in young patients with FMD were related to arterial dissection, with carotid arteries involved in 13% of cases.^3^

The best treatment of symptomatic patients with FMD and carotid tortuosity remains controversial and there are no randomized controlled trials comparing surgery vs. angioplasty in these conditions.^2^^,^^4^ Herein is reported the case of a young female patient, symptomatic for a TIA secondary to carotid artery dissection associated with kinking and FMD, who was successfully managed surgically by autogenous saphenous vein grafting.

## CASE REPORT

A 44-year-old female, with no past medical history, was referred to our institution two weeks after a history of aphasia, a motor deficit of the right upper limb, and left non-pulsatile headache with complete resolution after 2 hours of duration. For these symptoms, she was admitted to another center, where a cerebral magnetic resonance angiography (MRA) showed recent areas of infarction in the territory of the left cerebral parietal lobe. Moreover, the MRA was negative for carotid and vertebral artery stenosis or dissection and positive for left internal carotid artery (ICA) kinking and concomitant right ICA coiling. She was kept in neurological intensive care for three days and discharged with dual antiplatelet therapy (Aspirin 100 mg and Clopidogrel 75 mg per day).

**Figure 1:**
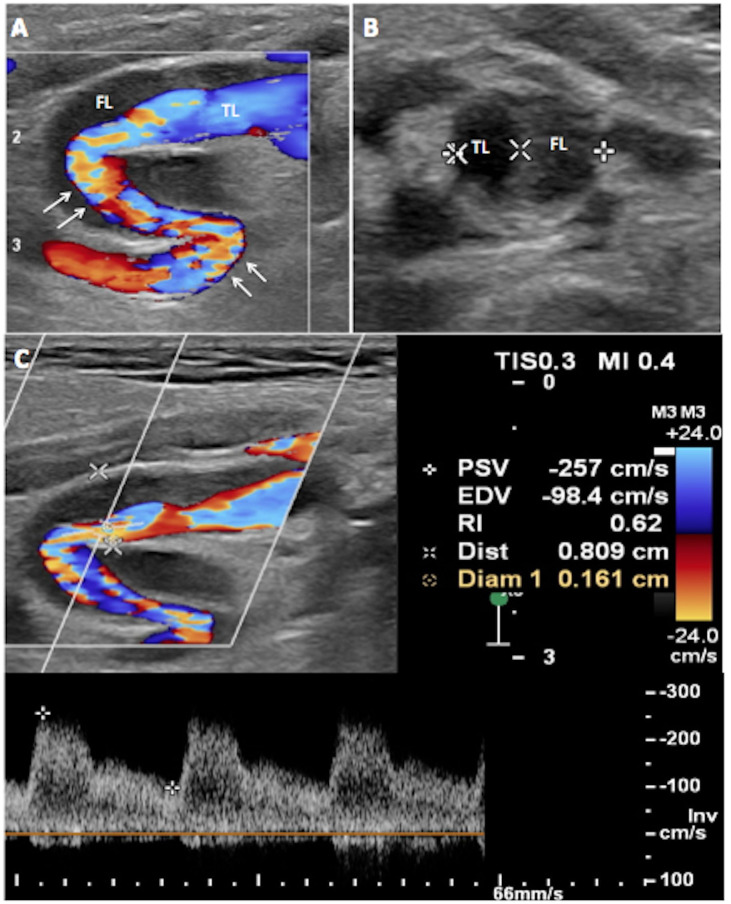
Preoperative Carotid Duplex Ultrasound. (A) There is turbulence (white arrows) in the left internal carotid artery (ICA) and tortuosity (S-curve morphology). The reduced arterial lumen caused by an arterial dissection is demonstrated, with false lumen (FL) and true lumen (TL); (B) Transverse view of the left ICA showing false lumen (FL) and true lumen (TL); (C) The spectral waveform shows markedly increased velocity of blood flow (PSV, peak systolic velocity 257cm/s) and presence of spectral bruit, indicating high-grade arterial stenosis.

On presentation to our vascular surgery outpatient clinic, the patient was neurologically asymptomatic with normal blood pressure control and no history of other neurological events after discharge. A duplex ultrasound (DUS) of cervical arteries was performed and revealed severe kinking of the left internal carotid artery, according to the Metz classification,^7^ with an important decrease in the artery diameter (> 70% of stenosis) and a probable image of ICA dissection with thrombosis of the false lumen (Figure 1). The patient was promptly admitted for recovery, scheduled for surgery, and a new brain magnetic resonance imaging (MRI) control was performed, showing no changes to the recent areas of infarct in the left parietal territory.

**Figure 2:**
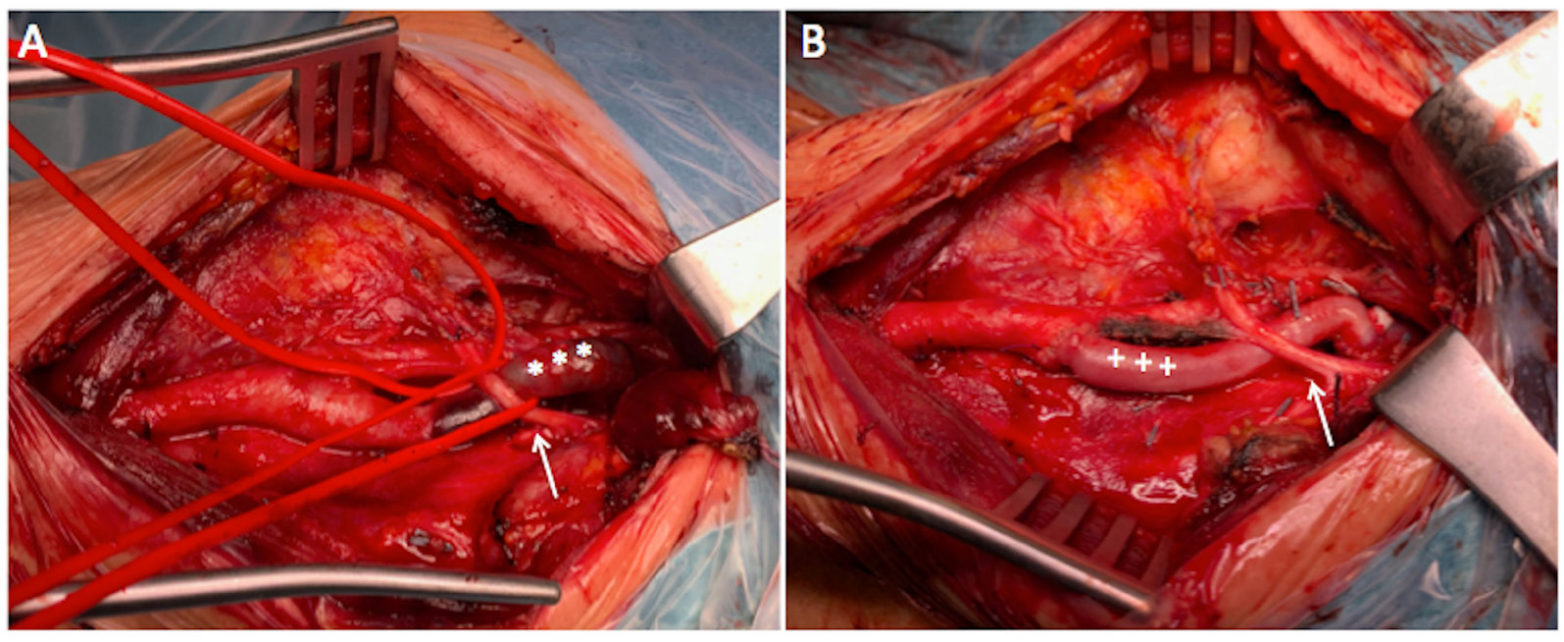
Intraoperative images. (A) Left carotid artery bifurcation showing dissection of the internal carotid artery with thrombosis of the false lumen (white asterisks); (B) Saphenous vein graft (white crosses) bypass from the carotid bifurcation to normal internal carotid artery. Hypoglossal nerve (white arrow).

Under general anesthesia, with nasotracheal intubation, electroencephalogram (EEG), and near-infrared spectroscopy (NIRS) for continuous non-invasive neurological monitoring, the patient underwent left pre-sternocleidomastoid cervicotomy incision to expose the extracranial carotid artery. After obtaining proximal and distal artery control, under systemic heparinization, the left common carotid artery (CCA) and distal ICA were temporarily clamped. After performing a short arteriotomy, a 9Fr Pruitt-Inahara Carotid Shunt (LeMaitre Vascular, Inc. Burlington, Mass) was inserted and no significant neurological changes were observed on NIRS or EEG monitoring. The arterial wall in the dissected and kinked segments appeared very thin, and resection of that area was performed. Reconstruction of the ICA was performed with a bypass using the autogenous reversed great saphenous vein (Figure 2). There were no perioperative complications, and the patient had an uneventful recovery. The patient was discharged home on the fourth postoperative day on dual antiplatelet therapy.

**Figure 3:**
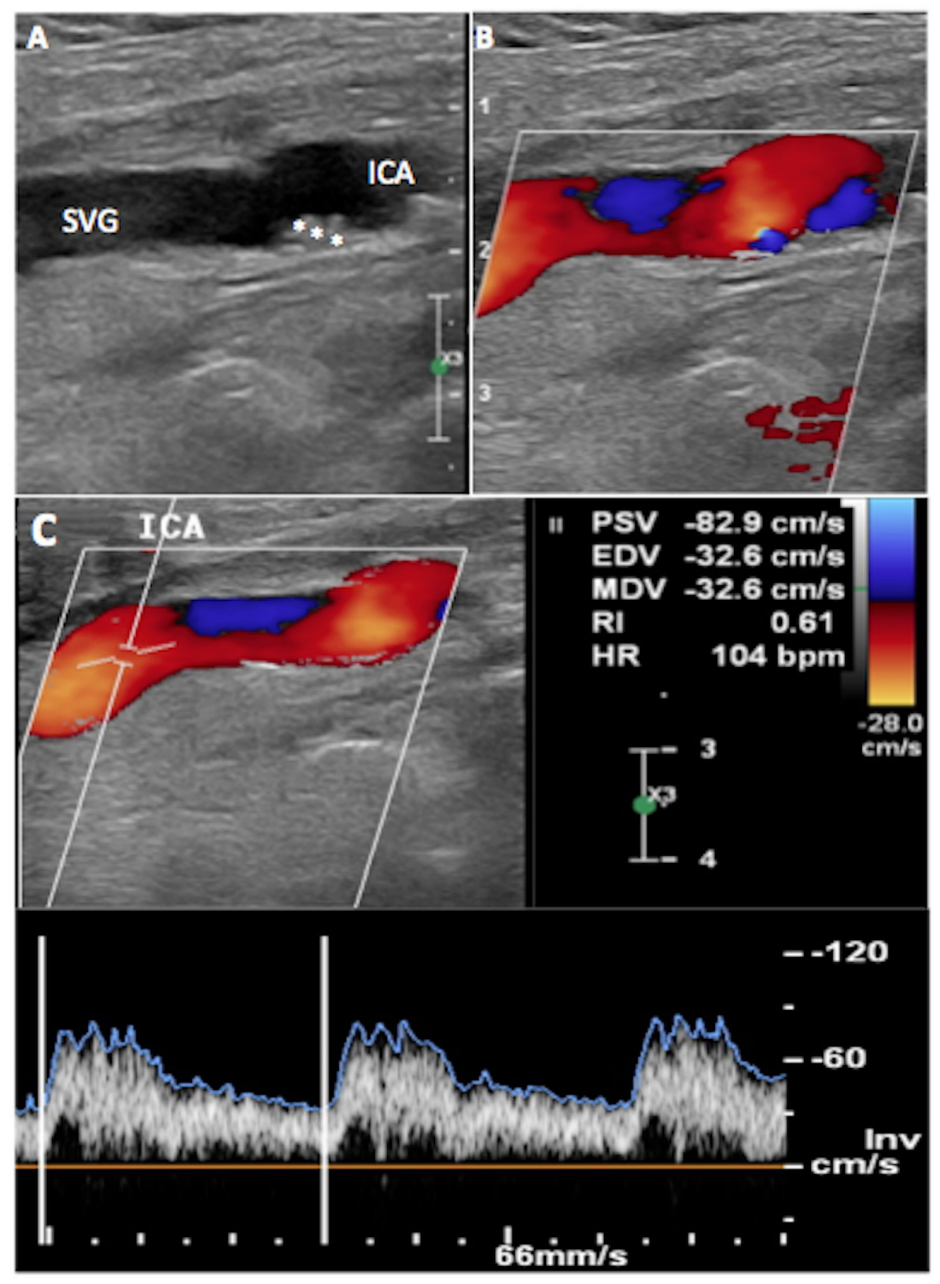
6-month post-operative carotid duplex ultrasound. (A) Saphenous vein graft (SVG) bypass to internal carotid artery (ICA). Anastomosis (white asterisks); (B) Patency of the SVG; (C) Spectral waveform showing normal blood flow velocity (PSV, peak systolic velocity 82.9cm/s).

**Figure 4:**
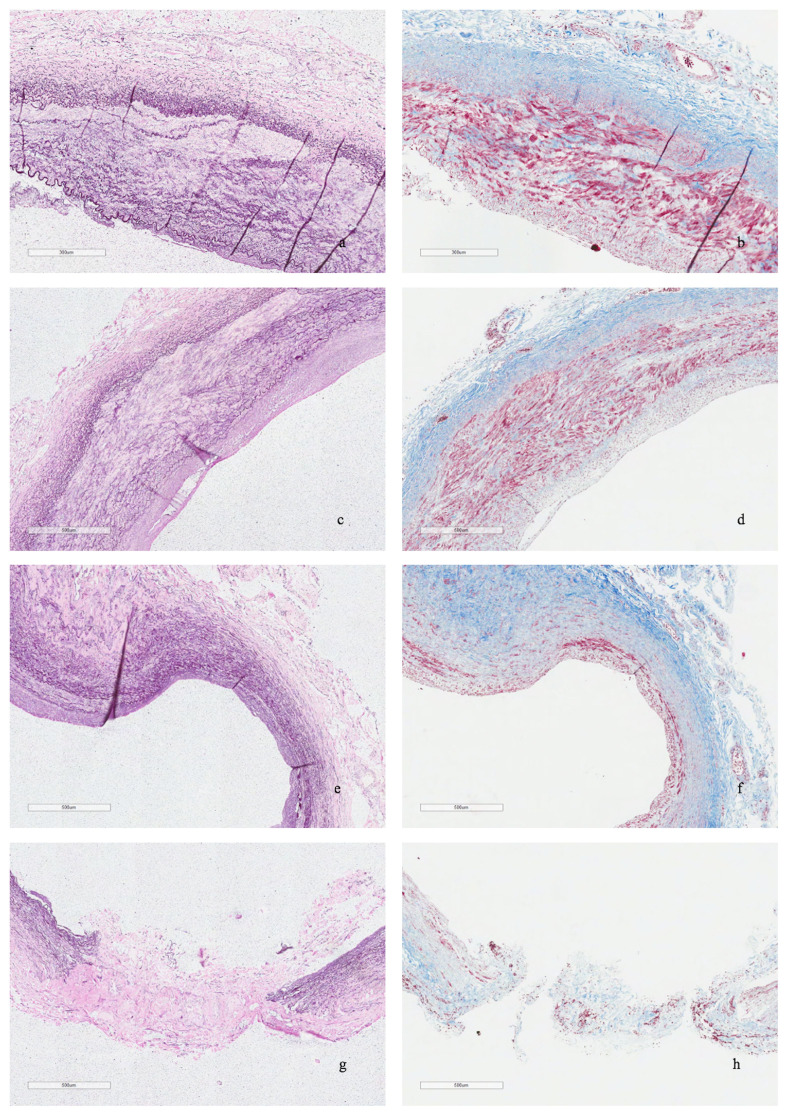
Histopathological evaluation. Elastic Van Gieson staining **(a, c, e, g)** and Masson trichrome staining **(b, d, f, h)** of carotid artery wall. Multifocal loss and fragmentation of elastic lamellae, severe disarray and loss of smooth muscle cells, leading to dilation and focal discontinuity of the media layer were observed. Minimal thickening and foamy macrophages were present in the intima layer. In **a, b, c, d, g,** and **h**: bar= 500 micron; in **e** and **f**: bar=600 micron.

At the 6-month follow-up control, the patient had no history of new neurologic events and DUS demonstrated a patent vein graft. (Figure 3) The resected segment of the ICA was sent to the Pathology Unit for histopathological evaluation, which reported ICA dissection, with focal loss of intima and media layers consistent with fibromuscular dysplasia (Figure 4). The patient consented to publication of this report.

## DISCUSSION

Carotid artery dissection is a well-known cause of ischemic stroke among young and middle-aged individuals.^8^ Although the pathogenesis of carotid dissections remains unknown, numerous risk factors have been postulated such as connective tissue disorders, infection, neck trauma, common neck movements, and atherosclerosis.^8^ According to many authors, FMD predisposes to ICA dissection, which may be the first clinical presentation.^3^^,^^4^^,^^6^^,^^9^ Even though the most common clinical manifestations of cerebrovascular FMD are headaches, pulsatile tinnitus, and dizziness, according to a US FMD registry, 1 in 5 patients experience a dissection and cerebrovascular events including transient ischemic attack, stroke, and/or amaurosis fugax occur in 1 of every 4 patients with FMD.^3^

Generally, the natural course of cerebrovascular FMD is benign and it is mostly found incidentally.^4^ Several imaging methods are useful for detecting irregular patterns of stenosis and aneurysms in cerebrovascular FMD. As FMD mostly affects the middle and distal portions of the ICA, cervical DUS has low sensitivity for detecting its typical patterns, such as turbulence and tortuosity. Nevertheless, DUS offers an accurate noninvasive method for detecting associated carotid artery lesions, such as dissection, significant stenosis, and presence of atherosclerotic lesions. According to Sethi et al.,^5^ a distinct morphological appearance of the mid to distal ICA visualized on carotid DUS, forming the shape of an ‘S’ (S-shaped curve), has a significantly higher prevalence in FMD patients as compared to control groups. Although not specific, the presence of this S-curve morphology should be interpreted as an alert to a possible diagnosis of FMD.^5^ Additionally, MRA should be performed to rule out the presence of intracranial aneurysms in patients with FMD, despite being an inaccurate method for detecting cervical artery injuries. In this case, the two methods were complementary and it could therefore be advisable to use more than one imaging method for correct diagnosis and management.

According to some authors, FMD might genetically predispose patients to dolichoarteriopathies of the internal carotid artery (DICAs), a group of anomalies that can be divided into tortuosity, coiling, and kinking of the ICA.^2^^,^^10^ Ballotta et al.^1^ histologically evaluated surgically treated carotid elongations and found that 56% of patients in their cohort had an FMD pattern. Paltseva et al.^11^ demonstrated that FMD patients present with impaired vascular elasticity properties due to destruction of elastic fibers and a decreased abundance of smooth muscle cells, which result in induction of tissue matrix degradation and could result in DICAs.^2^ The same histological pattern was observed in this case (Figure 4). These findings highlight the importance of correct FMD diagnosis, since this pathology involves multiple organs with increased risks of complications and death.^1^^,^^4^^,^^5^

After a suspicion or a definitive diagnostic of FMD, aggressive treatment of modifiable cardiovascular risk factors, such as hypertension, diabetes mellitus, and hyperlipidemia is supported and, in case of ischemic strokes, antithrombotic medications, including either antiplatelet therapy or anticoagulants are often administered.^6^ Assessment of the entire aorta and its branches with computed tomography (CT) or MRI is recommended for all FMD patients with strict annual DUS imaging surveillance unless new symptoms arise.^6^ According to Harriott et al.,^6^ patients with cervical artery dissection should avoid activities that increase the risk of sudden, rapid, or severe neck motion or that raise intrathoracic or abdominal pressure.

To date, there have been no randomized controlled trials demonstrating the best treatment for symptomatic patients with cerebrovascular FMD.^4^^,^^6^ According to some authors, symptomatic patients have a low rate of symptom recurrence or disease progression and, therefore, could be managed conservatively with stroke risk modification, antiplatelet agents, and surveillance imaging.^6^ On the other hand, presence of ICA dissection and kinking requires surgical or endovascular treatment.^2^^,^^4^^,^^6^ In recent decades, intraluminal angioplasty alone has been reported as the preferred treatment for FMD patients.^4^ However, the role of carotid angioplasty and stenting (CAS) in the management of severe ICA elongation in association with dissection is unclear. Whereas the endovascular approach has been described in a small cohort of patients with ICA dissection and tonsillar loop anatomy, it has not been reported in FMD patients.^12^ Moreover, the possibility of stent kinking due to the low radial force and vessel tortuosity could hinder catheter-guided procedures.

In this context, open surgery should be considered to treat the dissection and correct the ICA kinking or associated tortuosity. Surgical techniques include end-to-end anastomosis, end-to-side ICA reimplantation, eversion or patch carotid endarterectomy, and grafting of autogenous saphenous vein or patch grafting, with resection of the excess ICA.^2^^,^^4^ In addition, the long-term durability of ICA reconstruction after open repair is crucial, considering the young mean age of FMD patients. In the case reported herein, the fragility of the carotid artery wall due to the dissection made primary anastomosis unfeasible and, therefore, saphenous vein bypass grafting with artery shortening was chosen.

## CONCLUSION

Carotid artery dissection can be related to underlying pathologies such as FMD. In addition, the presence of ICA kinking and an S-curve pattern should be taken as an alert to the possibility of an FMD diagnosis. Surgical repair must be considered in symptomatic patients and histopathological evaluation is highly recommended to confirm the correct diagnosis and enable the best postoperative management.
